# Promoters and Detractors Identify Virtual Care as “Worlds Better than Nothing”: A Qualitative Study of Participating Veterinarians’ Perception of Virtual Care as a Tool for Providing Access

**DOI:** 10.3390/vetsci12020136

**Published:** 2025-02-07

**Authors:** Rosalie Fortin-Choquette, Jason B. Coe, Cathy A. Bauman, Lori M. Teller

**Affiliations:** 1 Department of Population Medicine, Ontario Veterinary College, Guelph, ON N1G 2W1, Canada; jcoe@uoguelph.ca (J.B.C.); cbauman@uoguelph.ca (C.A.B.); 2 College of Veterinary Medicine and Biosciences, Texas A&M University, College Station, TX 77843, USA; lteller@cvm.tamu.edu

**Keywords:** veterinarian, virtual care, access to care, semi-structured interviews, thematic analysis

## Abstract

(1) Background: Veterinary virtual care holds the potential to alleviate some barriers to accessing care, yet concerns within the profession exist. Understanding veterinarians’ perspectives and identifying the potential opportunities and challenges that virtual care poses for access to veterinary care are thus needed. (2) Methods: Semi-structured interviews were conducted virtually with 22 companion-animal veterinarians practicing across Canada and the United States. Interviews were accompanied by an electronic survey, with which a Net Promoter Score (NPS) was calculated for each participant. Using their NPS, participants were categorized as a “promoter” or “detractor”, with respect to their perspective on veterinary virtual care. A thematic analysis was conducted on verbatim transcripts of the interviews. (3) Results: A total of 11 detractors and 11 promoters were interviewed. Four subthemes were identified, including the following: (1) there are limitations to virtual care, (2) virtual care plays a role in access to care, (3) “virtual care is better than no care” and (4) virtual care offers specific value in supplementing in-person care. (4) Conclusion: When no other option for care delivery exists, virtual care was viewed as a way to increase access to veterinary care.

## Simple Summary

Virtual care offers a potential tool to increase access to veterinary care for pet owners. However, there are restrictions inherent to providing veterinary care remotely. To gain a better understanding of veterinarians’ perspective on the matter, interviews were performed with veterinarians holding opposing views with regard to virtual care. The interviews revealed that while virtual care has limitations, participants considered it useful in contexts where in-person care was not possible. Therefore, almost all participants considered virtual care to be “better than no care”, regardless of their viewpoint toward the remote delivery of care.

## 1. Introduction

In recent years, the integration of virtual care platforms in both human and veterinary medicine has been fast-tracked by the COVID-19 pandemic [1,2,3]. In the veterinary world specifically, public health lockdowns and occupational health concerns necessitated the widespread implementation of virtual care services. Moreover, the disruptions in day-to-day efforts and the changes in care delivery methods faced by veterinary teams created a backlog of wellness appointments, leaving a large number of pet owners unable to receive care in a timely manner and exacerbating access-to-care issues [4,5]. This new reality, coupled with the workforce shortage impacting the veterinary profession in Canada and in the United States [6,7], creates a need to adopt supplementary models for delivering veterinary care, such as virtual care. Therefore, in the United States and in Canada, veterinary associations have supported the appropriate use of virtual care to “augment the delivery and availability of high-quality veterinary care” [3] and to “fill some of the in-person gaps that currently exist in veterinary medicine” [8]. With that said, virtual care has yet to be accepted across the profession [9], and there is a need to understand in greater depth veterinarians’ perception of such a model for delivering care.

A recent survey in the United States identified virtual care to be increasingly welcomed by responding veterinarians during the pandemic, specifically when it was used to address the access-to-care issues that the spread of COVID-19 brought forward [5]. Reduced access to care is indeed a significant problem and has been identified as a current “animal welfare crisis” in the United States [5,10,11]. In Canada as well, a large pool of pets (roughly five million) does not receive veterinary care each year [12]. It has been found that millions of pets do not receive appropriate care due to accessibility issues such as financial, knowledge-based, geographic and/or demographic barriers [11]. However, as much as the supplemental use of virtual care can be viewed to alleviate some of the problems faced by the profession, some veterinarians remain uncertain about its validity [2]. Although research in the field of virtual veterinary medicine is still limited, a cross-sectional study published in 2022 reported that participating veterinarians were “extremely unwilling to recommend virtual consultations” to colleagues, and that they were less confident in reaching a diagnosis using virtual care [9]. Caution from veterinarians with regard to virtual care has in fact been documented in the current veterinary literature, alongside a negative perception of the level of care which can be provided remotely [2,9,13,14].

This perceived dichotomy within the veterinary profession, where some veterinarians express a greater interest in virtual care and others take caution in delivering care virtually, suggests the need to gain a better understanding of veterinarians’ perception of virtual care, and to identify the challenges and the benefits that the implementation of such models of care can bring to the profession. As one part of a larger study, the findings reported here provide a further understanding of veterinarians’ perception of virtual care with regard to providing access to care, including perspectives from veterinarians who hold opposing attitudes with respect to the use of virtual means for delivering veterinary care.

## 2. Materials and Methods

This study was approved by the University of Guelph Research Ethics Board (REB# 21-06-031, approved 17 September 2021). The Consolidated Criteria for Reporting Qualitative Research (COREQ) reporting guidelines for interviews and focus groups were followed.

### 2.1. Study Design

Recruitment involved purposive and snowball sampling to access the target characteristics (a positive/negative perspective of veterinary virtual care) within the study population [15]. The research team contacted multiple veterinary organizations with recruitment material and shared the study on social media platforms. Participants were also encouraged to share the study with their colleagues. In the recruitment material was a link to a Microsoft Bookings page (1.5.00), where interested veterinarians could book an online interview with the principal author (RFC). Participation in this study was open to licensed companion-animal veterinarians practicing in Canada or in the United States. Upon the booking of an online interview, an electronic questionnaire, which included a consent form, demographic questions and general questions pertaining to the individual’s perception of virtual care, was sent to participants to be completed ahead of their interview date. Interviews were conducted in English or in French, via Microsoft Teams (1.5.00) or by phone, between June and August 2022. An incentive to participate was provided by way of a CAD 50 Amazon gift card, which was sent electronically following the interview.

### 2.2. Questionnaire

The electronic questionnaire, which participants had to complete prior to the interview date, was constructed using the Qualtrics platform (Provo, UT, USA), was distributed via a personalized link and was used to collect the following demographic information: gender (male, female, my gender identity is not listed above or choose not to respond), age (18 to 25, 26 to 35, 36 to 45, 46 to 60, or 61 and over), year of graduation from veterinary college (open text box), country and state/province/territory in which they currently practice veterinary medicine (open text box), type of practice at which they work (corporate practice, a privately owned practice, or other with please specify), role in their current practice (owner/partner, associate, boarded specialist, locum, or other with please specify), average number of hours worked per week (open text box) and, finally, types of animals treated (companion animals, exotic animals, equine, food animals or other with please specify).

The survey also included a question to assess participants’ attitudes toward virtual care, based on which a Net Promoter Score (NPS) was calculated for each participant, with the question being as follows: “On a scale from 0–10, how likely are you to recommend the use of virtual care/telehealth to a fellow veterinarian?” Participants were identified as “detractors” if their score was from 0 to 6, “passive” if their score was 7 or 8 or “promoters” with regard to virtual care if their score was 9 or 10 [16]. The NPS is a validated tool to evaluate one’s perception of a product and has already been used to identify promoters and detractors in relation to virtual care in veterinary medicine [9].

### 2.3. Interview Guide

A semi-structured interview guide constituting six primary questions and numerous prompts and probes was formulated based on the current scientific literature relating to veterinary virtual care and access to care [9,13,17] (Appendix A). As the interviews were held during the COVID-19 pandemic, a definition of virtual care was provided to all participants at the beginning of their interview, to ensure their answers were not influenced by lockdown-specific measures: “We consider remote, or virtual care, to be any consultation where the client and their pet are not in the room with you. For the purpose of this study, we do not consider curbside drop-off to be virtual care.” Three pilot interviews were conducted with colleagues of the research team, who were veterinarians, to assess the feasibility and utility of the electronic questionnaire and discussion guide.

At the time of data collection, the principal author (RFC) was a PhD candidate studying epidemiology, with a research focus on veterinary virtual care, who had previously worked in veterinary clinics, with years of experience as a primary care giver for pets. RFC, who was the interviewer, adopted an intentionally curious, dispassionate and nonpartisan stance throughout the interviews. During the interview sessions, notes were taken by the interviewer, and at the end, an oral summary of said notes was offered to participants to seek validation. All interviews were recorded with the Microsoft Teams (1.5.00) integrated feature, and recordings were later roughly transcribed using the transcription function offered by Microsoft Streams (1.5.00) and reviewed by RFC to produce verbatim transcripts. Memoing was performed throughout data collection and transcription to optimize reflexivity.

### 2.4. Analysis

A thematic analysis [18] of the transcripts was performed by RFC using NVIVO (1.7.1). Themes were recognized by following patterns emerging from the data, and participants’ NPS was used to consider differences and similarities between virtual care promoters and detractors. The analysis followed a hybrid approach of inductive and deductive coding, where most themes were generated without any preconceived notions, but others were data-driven (for example, the theme “access to care” was also explored by asking a question at the end of the interview that included a definition of access). Sections of transcripts were selected based on the richness of the exchange and discussed between the principal (RFC) and second author (JBC) to support the rigor and interpretations of the thematic analysis.

## 3. Results

### 3.1. Participants

Eleven interviewed participants were categorized as promoters, seven as detractors and four as passive. During the analysis, passive participants were included in the “detractor” group, as the themes emerging from their interviews were similar. A total of 11 detractors and 11 promoters were thus considered during the thematic analysis.

In total, ten participants learned about the study from social media posts, four participants saw it shared via their telemedicine organization, four participants learned about the study via a provincial veterinary medical association newsletter and four participants were told about the study by their colleagues.

Of the 22 veterinarians interviewed, 5 identified as male and 17 identified as female. One participant opted for the interview to be conducted in French. The year of graduation ranged from 1984 to 2021. Seventeen participants were practicing in Canada and four in the United States, and one participant was practicing in both countries. Canadian provinces and territories where participants practiced included British Colombia (2), Ontario (13), Quebec (1), Yukon Territory (1) and Saskatchewan (2). States included Louisiana (1), Texas (1), Ohio (1) and Mississippi (1). All results from the questionnaire are reported in Appendix B.

### 3.2. Thematic Analysis

Data saturation, where no new information emerged, was reached before the end of the analysis of the 22 interviews. A total of eight overarching themes emerged from the interviews, including the impact of virtual care on access to care, on medical care provided, on client experience, on veterinarian–client communication, on day-to-day operations, on the system/structure surrounding the integration of virtual care models, on wellbeing and on the financial aspects of veterinary care. The overarching theme of virtual care’s impact on access to care was selected for this paper because both promoters and detractors highlighted, in agreement, that virtual care, although with limitations, can increase access to care. Four subthemes were identified, including the following: (1) there are limitations to virtual care, (2) virtual care plays a role in access to care, (3) “virtual care is better than no care” and (4) virtual care offers specific value in supplementing in-person care.

#### 3.2.1. There Are Limitations to Virtual Care

A prominent limitation and disadvantage that was raised by almost all detractors (nine) and promoters (eight) was not being able to perform a hands-on physical exam. Many detractors (five) as well as promoters (three) stressed that relying heavily on the clients’ description of the problem is a limitation of virtual interactions, which brings an element of uncertainty when formulating a diagnosis or giving advice. Likewise, two participating veterinarians (one detractor and one promoter) mentioned the fact that the visual component of a virtual interaction may not be optimal in relation to conducting the physical exam. Similarly, another detractor (one) expressed concern that the quality of care can be suboptimal, specifically because it is harder to “read the pet”. However, of the participants stating that the inability to perform a physical exam was a disadvantage to virtual care, many (five promoters and four detractors) acknowledged finding ways to work around this limitation:

“Pet is moving everywhere, the client is stressed, and they are often on their laptop which is not very flexible in terms of what they can show you, so I think the physical exam is definitely limited. I don’t disagree with that, but I’ve found ways around it to improve it and do the best that I can.” P101—Promoter

Following this line of thought, according to almost all promoters (nine) and some detractors (three), what can be achieved virtually depends heavily on a veterinarian’s level of comfort with performing remote consultations. From this perspective, one promoter described self-imposing guidelines, which, for this participant, included the following: “above all, do no harm”.

“You have to be smart about how you do it and there are of course some things that you have to say no to. So, I have very specific criteria that outline what can be seen and what can’t be seen […]. I know my limitations.” P100—Promoter

In association with not being able to perform a hands-on physical exam, some promoters (four) and detractors (two) also highlighted the fact that performing diagnostic testing was not possible with virtual care. A few participants (one promoter and two detractors) also acknowledged that not every situation can be addressed virtually, even if the only way for a pet owner to come in contact with a veterinarian is via virtual care:

“It is certainly very difficult for people to have pets and livestock in remote communities. How do you get them a rabies vaccine? What if the dog has a GDV [Gastric dilatation-volvulus]?” P142—Detractor

Similarly, a disadvantage raised by a single detractor was that some platforms may complicate the continuity of care by not allowing a care provider to stay in touch with the client after a “one-off” virtual interaction. This participant gave the example of a telemedicine platform that would allow a veterinarian to offer a consultation to a client far away from them, and then posed the question of how they would offer care if subsequent interventions were needed. In addition, one detractor mentioned that access can be bound by the fact that some jurisdictional legislations keep veterinarians from reaching a broader territory. For example, this participant, practicing a specialty in Canada, mentioned not being allowed to offer virtual consultations to clients beyond their province’s borders, even if their service was needed, limiting the opportunity for broader access to care through virtual means.

A couple of participants (one promoter and one detractor) highlighted the fact that pet owners seeking help virtually may delay care if nothing can be achieved remotely to help the animal. For example, in the context of an emergency necessitating in-person interventions, the time spent in a virtual consultation could have been spent driving to a brick-and-mortar clinic. Adding on to this thought, one of these participants shared their own experience with human healthcare (where seeking help via telemedicine can delay care as well), which shaped their opinion of virtual veterinary care:

“Telehealth is so limited—in human medicine too. […] I’ve used telehealth many, many times over the years. What have they invariably said? Go to the doctor. […] I don’t even know why I called. I should have just gone [in person].” P119—Detractor

Some participants (one promoter and two detractors) also mentioned that there can be added distractions with virtual care. Given as an example by a participant, a pet owner could be taking the call from their car or be in any other situation that may affect their ability to concentrate. A few promoters (three) pointed out the possibility of tech issues, which may also derail a consultation. Another detractor (one) raised the point that virtual interactions remove the social aspect from veterinary medicine, which can impact relationship building and the perceived value of the service provided, given that some clients value human contact:

“That social aspect is… is huge. [Clients] want… they want somebody who’s gonna love their dogs the way they do.” P139—Detractor

#### 3.2.2. Virtual Care Plays a Role in Access to Care

Although several disadvantages to virtual care were brought forward during the interviews, the overall perception that virtual care could be a tool to increase access to care, especially when geographical restrictions exist, was conveyed by almost all participants (eleven promoters and nine detractors). A handful of promoters (three) also mentioned that virtual care can facilitate access to specialists for owners when geographical barriers exist.

Almost all participants (ten promoters and nine detractors) mentioned increased financial accessibility offered by virtual consultations. The discussion surrounding the financial aspects of virtual care was broad, but increased accessibility with regard to socioeconomic status was mentioned specifically with regard to the possibility of offering remote care at a lower cost compared to in-person care. For instance, some participants mentioned the fact that some pet owners may only be able to afford a consultation fee and feel more comfortable intentionally arranging and paying for a virtual consultation knowing that they will not be required to have diagnostic tests performed on their pet immediately. Other participants mentioned offering virtual consultations at a lower cost compared to in-person consultations. Yet, in contrast, a few promoters (four) and detractors (two) pointed out that for them, financial accessibility by reason of the cost of a virtual consultation being less was a moot point because, in their experience, there is minimal difference between the price of a consultation at brick-and-mortar practices and the price on most virtual platforms.

According to some participants (two promoters and one detractor), virtual care can also increase overall accessibility as it can help care providers and their clients stay connected more often by facilitating increased touchpoints. For example, one promoter mentioned that digital means (emails, for example) can be used to communicate with clients in terms of preventive care initiatives (e.g., vaccination, deworming, etc.). Many promoters (five) and detractors (four) similarly mentioned that virtual care, in allowing veterinary professionals to be more connected and accessible to clients, could facilitate client education and break down knowledge-based barriers to accessing veterinary care. Participants provided examples of using virtual care to provide supplementary material to clients, such as videos kept in a bank of digital resources, or adding virtual touchpoints to their face-to-face interactions, where they provide additional information or clarify more complex concepts brought up in person:

“If they’ve gone to their regular vet and the cat’s been diagnosed with diabetes and, you know, it’s overwhelming to get all that information all in a big pile at once in an exam room, and the cat is there and, you know, they get home and they’re like: ‘gosh, I’m not sure I quite understand this’, you know. Then telemedicine could be there to help. You know, clarify things.” P133—Promoter

Additionally, several detractors (five) and one promoter mentioned the fact that virtual care options contribute to reducing the challenge of “Dr. Google” by offering more easily accessible advice from professionals and “opening the field to more pet management discussions” with pet owners (P103—Detractor).

“[Virtual care platforms] make it easier for a client that would have otherwise probably just […] diagnosed their dog on Dr. Google and sought treatment on Amazon. So, I think by making access to a vet more accessible, it makes physical care more accessible as well.” P102—Promoter

Although the overall view of participants was that virtual care offers a way to increase contact points with veterinary professionals and reduce misinformation, one promoter and one detractor shared their view that in-person interactions are needed to establish an initial connection, to then provide education opportunities via virtual care. For example, one of these two participants, when talking about their work in remote communities, specified that in-person interactions were crucial to provide the necessary education for the pet owners “to then understand the value of vaccinations and seeing a veterinarian [every year]”. (P131—Detractor)

A current constraint to increasing access to veterinary medicine through virtual care raised by one promoter and one detractor was the expressed perception that the public is not aware of the veterinary services that can be offered virtually. These participants felt that current virtual care platforms are underutilized for this reason, limiting the access that could be offered by virtual care. In addition, divergent views were expressed regarding pet owners’ access to technology, with several promoters (two) and detractors (four) bringing up the fact that technology and devices used for virtual care are not available for all. In contrast, two promoters and one detractor indicated that technology is nowadays readily available for the majority of the population, as described by one participant practicing veterinary medicine in northern Canadian communities:

“Um, most communities—I mean, most people have Internet. Facebook. Most people have that and most of the communities, if they don’t directly have it, either their friends have it, or they have it available at public libraries.” P131—Detractor

Other mentions were made by participants in relation to the theme of access to care. Notably, a few promoters (three) and one detractor mentioned that virtual options for care increase accessibility for people with physical limitations (pet owners with mobility issues, for example), and one promoter pointed out that virtual care can also help clients who do not have means of transportation. In addition, one promoter pointed out that virtual care can increase access to some medication by offering a prescription via telemedicine that can be filled at a local pharmacy. One promoter also pointed out that seeking in-person care could sometimes lead to embarrassment for pet owners that fear being judged (based on physical appearance, for example) and that virtual options may reduce this barrier.

“Sometimes people are too embarrassed to get help. […] They don’t want to come to a hospital situation because they’re afraid they’re going to get judged […] Whereas virtually, it’s just a conversation [they] can have in [their] safe environment—whatever that is—and [they are] more comfortable. And again, it’s a win for them.” P100—Promoter

#### 3.2.3. “Virtual Care Is Better than No Care”

While many participants (six detractors and three promoters) expressed a view that face-to-face care remains best practice in veterinary medicine, several promoters (eight) and detractors (seven) explicitly concluded that virtual care was a viable alternative for clients who could not obtain in-person care, indicating that “virtual care is better than no care”. Although not mentioned explicitly by all participants, it was an underlying view evident in all interviews that virtual care is a tool that can be used to increase access to veterinary care by allowing the ability to do more than nothing.

“It’s kind of an all or nothing situation. From an ethics and welfare standpoint, it would be better to perform a physical exam, but when you’ve got a barrier that’s absolutely preventing you from doing that, then I think virtual care is worlds better than nothing.” P103—Detractor

“At least, you know I—from my perspective at least, something is being done rather than… an animal just suffering and, and nothing being done. So there, I mean, there is a positive side, yeah.” P131—Detractor

The idea that “virtual care is better than no care” was present across many topics, including the affordability of care. Two participants (one promoter and one detractor) mentioned that virtual care is “better than no care” in contexts where pet owners cannot afford the potential costs associated with in-person care, yet others (two detractors and one promoter) cautioned that money should not be the motivation to provide care which could potentially fall below what can be offered in person.

“I do still think that in-person exam and interaction is very, very important. And I wouldn’t want to see it replaced by virtual care just because virtual care might be able to be less expensive for someone.” P130—Promoter

A couple of participants (one promoter and one detractor) also indicated that building rapport and establishing a VCPR in person is favorable, but that in contexts where nothing else is available, it is possible to establish a relationship virtually. In addition, a few participants (one promoter and two detractors) also suggested that virtual care was “better than nothing”, specifically in contexts where brick-and-mortar clinics are overloaded and cannot meet the scheduling needs of clients.

A couple of detractors (two) and, similarly, promoters (two) indicated that the value of virtual care lies within the ability to provide advice and guidance to pet owners when no other action is possible. In their opinion, it is because when in-person care cannot be sought (because no emergency clinics are within reasonable driving distance, for example), professional advice can still be beneficial to pet owners (as per a participant, to alleviate anxiety). According to the two detractors mentioned above, virtual care can therefore be valuable in offering comfort to pet owners. Incidentally, a few participants (two promoters and one detractor) shared their common experience of pet owners feeling grateful for being seen virtually by a professional when no other option was available, even when said clients were aware of the limitations of a remote consultation:

“I mean, I know the client would much prefer to have the animal with me, you know, to actually do a proper physical exam. […] But, usually, they’re just appreciative to be getting any help at all.” P131—Detractor

#### 3.2.4. Virtual Care Offers Specific Value in Supplementing In-Person Care

As mentioned, most participants expressed the view that in-person care remains the best-practice option. Despite this underlying view, many promoters (four) as well as detractors (three) expressed that virtual care holds specific value in the realm of veterinary medicine when it is viewed as supplemental to in-person care. A couple of promoters (two) and a detractor (one) specifically indicated that virtual care becomes valuable when it is used as a stopgap before a pet owner can see a veterinarian in person.

“There are times where I can’t treat the whole situation, but I can still make an improvement and buy them a bit of time, perhaps until they can go to their regular clinic” P133—Promoter

A few participants (two promoters and one detractor) indicated that virtual care can also be a beneficial tool to help pet owners locate brick-and-mortar clinics that may be available to them, specifically in emergency contexts. Others (one promoter and one detractor) mentioned the fact that virtual care can be used to inform clients of when to access in-person care or to provide health markers that are clear signs, indicating that the situation has become an emergency and that in-person care should be pursued.

“[…] there have been a few times where I have said: I honestly can’t do much for you other than talk you through this and tell you that yes, you need to go to the [emergency] clinic. And they are still thankful at the end because I have given them direction and confirmed what they thought they had to do. So, I think it is very valuable, and clients seem to think so too, from what I have heard.” P133—Promoter

## 4. Discussion

The findings of this study provide a deeper understanding of participating veterinarians’ views on the role that virtual care can play in accessing veterinary care. Most participants raised limitations regarding the remote nature of virtual care, yet virtual care was, in general, viewed by participants to have an important role in increasing access to care. Across participating veterinarians, whether promoters or detractors, the implicit theme that “virtual care is better than no care” was clear.

The inability to access care is a subject of increased concern in veterinary medicine, as one out of four pet owners in the United States have been reported to experience barriers in obtaining veterinary care [19]. In Canada, similar access-to-care issues are noted, as a recent survey identified that 18% of responding pet owners could not access preventative care, and 12% could not access sick care for their pet [20]. The current literature describes the most common barriers to veterinary care as financial, geographic (which commonly includes location and transport), demographic (culture and language, for example), and knowledge-based, which relates to client education [7,17,21]. According to Neal and Greenberg (2022), access to veterinary care is a multifaceted issue that “sits at the intersection of a number of societal factors including income inequality, access to transportation, language and cultural differences as well as the spatial distribution of veterinary care providers” [22]. Specifically, a recent scoping review found that financial- and geographic-related issues were the two most reported barriers to accessing veterinary care in the United States and in Canada [23], two prominent areas that emerged from interviews with participants of the present study in relation to the potential opportunity virtual care offers in helping address access-to-veterinary-care barriers.

Almost all participants in this study, detractors similarly to promoters, mentioned the possibility of increasing geographical access to veterinary care by means of virtual care. This is consistent with the current literature, where the use of telemedicine has been reported to help overcome geographical barriers for veterinary clients [5,24]. Geographical barriers to care pose both logistical (e.g., delivery of medical supplies) and sociocultural (e.g., cultural beliefs surrounding the need for preventative care) challenges for veterinary medicine [17]. Participants in the present study shared that virtual care may be able to address some of these challenges by improving access to care from a logistical standpoint by adding channels of communication through which to reach out to clients in remote areas. Along with the importance of increasing access to underserved areas, additional geographic and demographic access issues were considered by a number of participants of the present study as having the potential to be addressed with virtual care (as an alternative when in-person interactions cannot be offered). The distance to a veterinary clinic from a pet owner’s home, transportation and the overall inability of a pet owner to physically access a veterinary facility are important barriers [22,25], and all three problems were touched on by participants in the present study as having the potential to be alleviated with virtual care. Therefore, virtual care provides another tool for veterinary professionals to consider in addressing geographical and demographic barriers to care that can be experienced by pet owners and their animals.

In addition to geographic and demographic access, most participating veterinarians considered financial accessibility when sharing their views on the role that virtual care has in increasing access to veterinary care. Client finances have been reported to be the most important barrier to care for veterinary clients, regardless of a pet owner’s income category [10]. While most participants viewed virtual care as a potential tool to help address cost barriers by offering services at a lower cost, some did not agree with this approach, explicitly conveying that virtual care should not be viewed as a replacement for in-person care for cost-related reasons. This tension and divergence of opinion identified among participants of the present study are important to note and may warrant further research into how virtual care can most effectively support increasing veterinary clients and patients’ financial access to care, while maintaining the sustainability of veterinary practice.

On a systemic level, geographic barriers are often tied to socioeconomic barriers [17,26], where geographic access issues may be exacerbated in low-income areas [10]. One could therefore view virtual care as an interesting tool to improve access to care when the intersection between financial barriers and geographical barriers exists. In fact, a recent study found that pet owners from vulnerable communities were significantly more likely to use telemedicine when compared to pet owners from less vulnerable communities [5]. Many participants of the present study viewed virtual care as having the potential to alleviate both financial and geographic barriers, however with many caveats, including the inability to perform a complete physical exam or offer treatments or procedures that require in-person delivery. Developing further research to explore pet owners from vulnerable communities’ perceptions with regard to veterinary virtual care may generate additional insight into how certain virtual care delivery models may be utilized to reduce access issues when both geographic and socioeconomic barriers exist.

Although not mentioned as often as geographic and financial barriers, the role of virtual care in addressing knowledge-based barriers to care emerged from the analysis of the present interviews, with virtual care mentioned by some participants as a way to promote or increase client education. A lack of awareness in terms of the benefits of routine veterinary care has often been found to be more predominant in vulnerable communities, and, in this regard, the current literature highlights the importance of educative and positive interactions from veterinary professionals in underserved areas [21]. Such interactions could be provided via virtual care (in supplement to in-person interactions), as suggested by participants of the present study, a tool that may help navigate logistical barriers to provide information to underserved communities, warranting further consideration by the veterinary profession.

In relation to the theme of improving access to care by improving client knowledge, some participants also saw virtual care as a chance to increase access to education and curb the problem of misinformation brought on by the Internet, often cited as a challenge for veterinary professionals [27]. Increased access to virtual platforms monitored by veterinary professionals may in fact reduce pet owners’ reliance on unverified sources. This idea is reinforced by previous findings, which report that veterinarians are the preferred source of information for pet owners [21,28]. Additionally, some participants in the present study mentioned virtual care as a way to provide supplemental education to clients, which aligns with the findings of recent qualitative studies that identified pet owners’ desire to have easy access to supplemental information provided by their veterinarians [27,28]. Continuing to explore how virtual care could be maximized as a tool to address client-identified needs and access to education may assist veterinary professionals in addressing client misinformation.

In all, the results from the present study suggest that virtual care may be a tool to navigate different access barriers to veterinary care delivery, including situations where different barriers coexist. In fact, access to care in the human healthcare industry has been broken down into five pillars, described as acceptability, affordability, availability, accessibility and accommodation [11]. As found by the present study, virtual care could be viewed as a way to improve accessibility via affordability, as there is a perceived notion that virtual care could allow for flexible and cost-effective options. Accessibility could as well be improved via acceptability, in providing additional educational tools and resources to pet owners. Virtual care could also provide accommodation and availability by improving physical and logistical accessibility and by offering flexible and convenient ways for veterinary clients and their animals to access care.

As much as virtual care seemed to be viewed positively by almost all participants of the present study (promoters and detractors alike) with respect to providing accessibility to veterinary care when no other option exists, the underlying notion of in-person care being the best way to deliver care, when possible, prevailed throughout the interviews. The lack of the possibility to perform a complete, hands-on physical exam in virtual consultations was a primary concern voiced by most participants, acknowledging that there are clinical contexts when, if possible, an in-person consultation should be prioritized or required. For instance, a recent survey found that veterinarians were least willing to use virtual care for a sick pet’s initial visit, for pre-surgery visits or for after-hours/emergency visits [9]. With the recognition that access barriers do exist and that virtual care offers an option for circumstances when no other options for care are available, further research is needed to develop best practices that support increasing clients and patients’ access to veterinary care through remote means, which includes continuing to develop an understanding of the virtual physical exam. Several participants in the present study expressed being able to work around some of the limitations, specifically with regard to the physical exam, which would expand the opportunity that virtual care offers to veterinary clients and patients in accessing veterinary care.

Virtual care was seen by most participants as a secondary option for veterinary care delivery which could not always offer veterinary patients and clients what is possible through in-person care. Concerns with misdiagnosis, inadequate treatment and liability are indeed recurrent themes in the current veterinary literature with regard to virtual care [2,5,9,13,14]. Yet, without compromising the quality of care provided to a client, a wide range of options is often available, and it is worth noting that providing only what one may consider to be the “best” treatment does not always meet the needs of a pet owner [29,30]. In this spirit, concerns with only following best practices can become a barrier to the accessibility of care [17]. Similarly, the view held by a few participants that virtual care should not be viewed as a replacement for in-person care for cost-related reasons has the potential to perpetuate financial access barriers to veterinary care. With this in mind, in situations where in-person care is not possible or financially accessible, virtual care may offer a context-specific alternative that effectively provides access to care for a pet owner and their animal. This was a notion expressed by several participants and is consistent with the ongoing discussion around approaching the delivery of veterinary care using a spectrum-of-care model. The term “spectrum of care” is increasingly predominant in the field of veterinary medicine, where the perceived best diagnostic or treatment by the veterinarian does not necessarily match the circumstances or resources of a pet owner, and, rather, the optimal approach is often variable and contextually dependent on the situation of the client and patient [25,30]. A key element to the practice of a spectrum of care is weighing the advantages and disadvantages of the available options in relation to the contextual requirements of a client and their animal. There are multiple factors which can influence the type of care that can be provided, and these factors include a pet owner’s circumstances and available resources [25,31]. In this regard, virtual care is another tool which allows the contextualization of care, as it can provide relief to pet owners unable to geographically or financially access in-person care, that is, even if in-person care remains best practice. Further research exploring the perspective of pet owners facing access issues could benefit the profession, as identifying their unique considerations and needs with regard to the remote delivery of care would provide further insight into the implementation of valid and sustainable models of virtual care delivery.

## 5. Limitations

Semi-structured interviews were performed to explore and allow a deeper understanding of participating veterinarians’ perspectives of virtual care. It is important to bear in mind that qualitative research is not hypothesis testing, but rather hypothesis generating. Hence, the present results may not be generalized to all veterinarians but provide valuable insights for consideration and offer leads for future research. Although the generalization of the results is not possible, efforts were made to interview companion-animal veterinarians from various backgrounds (provinces and states with different legislative requirements with regard to virtual care) with a wide range of opinions (detractors and promoters). In this regard, data saturation was reached, supporting the rigor of the findings among the population accessed for this study.

## 6. Conclusions

Virtual care was largely viewed by participants as a tool that can be used by veterinary professionals to improve access to care with respect to geographic, demographic, financial and knowledge-based access barriers. Future research exploring pet owners’ perspectives of virtual care as a tool to improve access to care may provide a further understanding of the gaps that could be filled by virtual care. While virtual care offers a way to combat access-to-care issues, especially when other care options do not exist, uncertainty remains with regard to the quality of care that can be provided virtually, as expressed by most participants of the present study. Best practices in relation to virtual care should be explored, with guidance offered to the profession to facilitate and support, when appropriate, a hybrid model for delivering veterinary care.

## Data Availability

The interviews used for this article are not available because of ethical restrictions. All relevant data are provided within the paper.

## Appendix A. (Discussion Guide)

**Table A1:** Interview guide.

Primary interview questions
*First of all, can you tell me, what does virtual care mean to you?*
*Now, looking back at your experience in the veterinary profession, what are your thoughts on how virtual care compares to face-to-face visits?*
*Virtual care has been used more and more recently. How has the increased use of virtual care affected day-to-day operations at your workplace?*
*What are your thoughts on the current regulations that exist with respect to virtual care?*
*What are your thoughts on the economic impacts or the potential economic impacts of virtual care on a veterinary practice?*
*What are your thoughts on the role of virtual care in veterinarians’ well-being?*
*I’d like to now spend a few minutes discussing access to care, but to start, I would like to clarify how we define “access to care” in the context of this study because access to care is a broad term that can be defined many ways. Based on a 2019 paper by Dr. Michelle Lem, where 3 barriers to accessible care are considered:* *1. Socio-economic* *2. Geographic* *3. Knowledge-based* *With this in mind, what do you believe are the opportunities for virtual care to improve access to veterinary care* - *On a socio-economic standpoint?* - *On a geographic standpoint?* - *On a knowledge-based standpoint? (Knowledge-based barriers can be reduced through education, communication, population control, public engagement, etc.)*
*To conclude this discussion, I would like for you to look back at your experience with virtual care in the veterinary profession. Throughout this discussion you have highlighted a lot of examples where you thought telehealth worked or didn’t work. Is there anything you would like to add?*

## Appendix B. (Electronic Survey Results)

**Table A2:** Demographic data (general, *n* = 22).

GENDER	
FEMALE	17
MALE	5
AGE	
26 TO 35	5
36 TO 45	12
46 TO 60	3
60 AND OVER	2
YEAR OF GRADUATION	
MIN	2022
MAX	1984
MEAN	2007
MEDIAN	2008
COUNTRY OF PRACTICE	
CANADA	18
USA	3
CANADA AND USA	1
PROVINCE/TERRITORY/STATE	
BC	2
ON	13
QC	1
SK	2
YK	1
MISSISSIPPI	1
OHIO	1
LOUISIANA	1
TEXAS	1

**Table A3:** Demographic data (practice-related, *n* = 22).

TYPE OF PRACTICE	
CORPORATE	4
PRIVATELY OWNED	14
TELEMEDICINE PLATFORM	1
ACADEMIA	3
FEDERAL GOVT.	1
ROLE WITHIN THE PRACTICE	
OWNER/PARTNER	8
ASSOCIATE	10
Other, please specify:	
RESIDENT	1
FRONT LINE VETERINARY INSPECTOR	1
VICE PRESIDENT OF VETERINARY LEADERSHIP	1
PROFESSOR	1
HOURS WORKED PER WEEK	
MIN	26
MAX	60
MEAN	39
MEDIAN	36.5
TYPES OF ANIMALS TREATED	
COMPANION ANIMALS	22
EXOTIC	4
EQUINE	2
FOOD ANIMALS	3
Other, please specify	
BEES	1
BACKYARD POULTRY	1
ANIMAL PRODUCTS/BY-PRODUCTS	1

**Table A4:** Attitude toward virtual care (*n* = 22).

CONFIDENCE WITH TELEMEDICINE (on a scale from 1 to 100)			
MIN	10/100		
MAX	100/100		
MEAN	68.045/100		
MEDIAN	81/100		
COMFORT WITH TECHNOLOGY (on a scale from 1 to 100)			
MIN	31/100		
MAX	100/100		
MEAN	77/100		
MEDIAN	81/100		
COMMUNICATION TOOLS USED MOST IN PRACTICE (place in order)			
First choice		Last choice	
Phone	13	Video conferencing	8
Email	6	Sharing photos digitally	5
Asynchronous texting/messaging	1	Social media platform (ex. Vet advice groups on facebook)	3
Video conferencing	1	Synchronous/live texting/messaging	3
Social media platform (ex. Vet advice groups on facebook)	1	Sharing videos digitally	2
		Asynchronous texting/messaging	1
CONFIDENCE IN REACHING A DIAGNOSIS IF			
Hands on examination of the patient and face-to-face interaction with the pet owner	MIN	61/100	
	MAX	100/100	
	MEAN	87.13/100	
	MEDIAN	90/100	
Hands on examination of the patient and virtual interaction with the owner (ex. patient has been dropped off at the hospital and the client does not enter the building)	MIN	62/100	
	MAX	100/100	
	MEAN	82.72/100	
	MEDIAN	85/100	
Virtual examination of the patient and virtual interaction with the client	MIN	20/100	
	MAX	94/100	
	MEAN	62.68/100	
	MEDIAN	66.5/100	
No examination of the patient and virtual interaction with the client	MIN	10/100	
	MAX	93/100	
	MEAN	42.68/100	
	MEDIAN	37.5/100	
